# The structure of first-cousin marriages in Brazil

**DOI:** 10.1038/s41598-020-72366-z

**Published:** 2020-09-23

**Authors:** Paulo A. Otto, Renan B. Lemes, Allysson A. Farias, Mathias Weller, Shirley O. A. Lima, Victor Alves Albino, Yanna K. Marques-Alves, Eliete Pardono, Magnolia A. P. Bocangel, Silvana Santos

**Affiliations:** 1 Departamento de Genética e Biologia Evolutiva, Instituto de Biociências, Universidade de São Paulo, Rua do Matão 277, São Paulo, SP 05508-090 Brazil; 2 Núcleo de Estudos em Genética e Educação, Universidade Estadual da Paraíba, Rua das Baraúnas, s/n - Prédio da Integração Acadêmica - sala 329, Campus I – Bodocongó, Campina Grande, Paraíba, Brazil

**Keywords:** Evolution, Genetics

## Abstract

This paper deals with the frequency and structure of first-cousin marriages, by far the most important and frequent type of consanguineous mating in human populations. Based on the analysis of large amounts of data from the world literature and from large Brazilian samples recently collected, we suggest some explanations for the asymmetry of sexes among the parental sibs of first-cousin marriages. We suggest also a simple manner to correct the method that uses population surnames to assess the different Wright fixation indexes F_IS_, F_ST_ and F_IT_ taking into account not only alternative methods of surname transmission, but also the asymmetries that are almost always observed in the distribution of sexes among the parental sibs of first-cousins.

## Introduction

**Figure 1:**
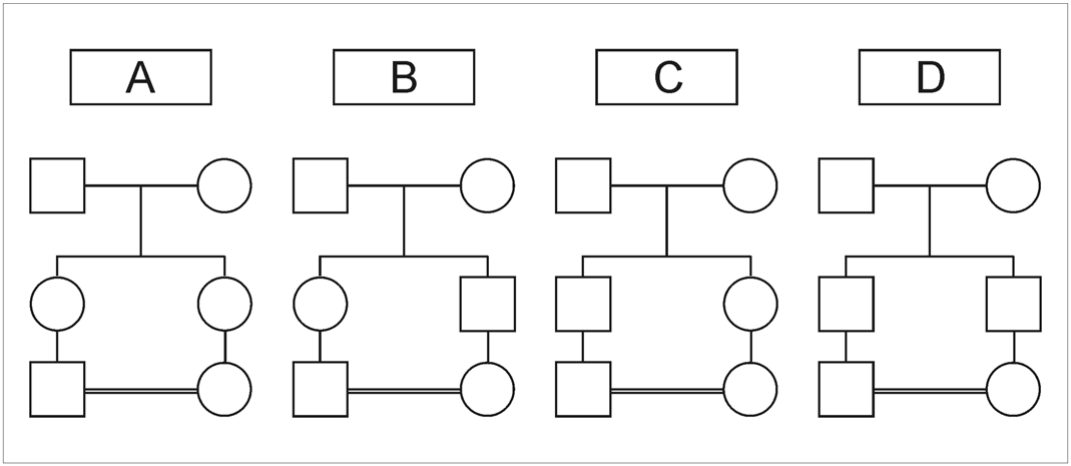
Subtypes of first-cousin marriages (see text for details).

The structure of first-cousin marriages has been the subject of several investigations starting some 60 years ago, with at least three of them performed in Brazil^1–3^. The first-cousin marriages can be classified into four subtypes, according to the gender of the two sibs parents of the couple of cousins, as shown in Fig. 1: in subtype A, the couple of first cousins are the offspring of two sisters; in subtype B, the mother of the male cousin is the sister of the father of the female cousin; subtype C is the inverse of subtype B; finally, in subtype D, the couple of first cousins are the offspring of two brothers. In some papers the subtypes A, B, C, and D are alternatively named 3, 4, 2, and 1, respectively. Subtypes A and B, for allowing occasionally the formation of X-linked autozygous daughters from the couple of first cousins, are also known in the literature by the name X-inbred, while subtypes C and D, for blocking the formation of autozygous female offspring, receive the name of X-outbred. It is interesting to note that almost always the observed relative frequencies of subtypes A, B, C, and D differ significantly from equal (1/4) expected population frequencies.

The population average probability of homozygosity by descent (autozygosity) is commonly estimated from the analysis of sets of genealogies and quantified by Wright’s fixation index F_IT_. This index takes into account not only cases of panmixia deviations measured by the fixation index F_IS_ but also cases in which the homozygosity is due to small population size and other random effects (quantified by the fixation index F_ST_). Because an individual cannot be homozygous due to both inbreeding and random effects, F_IT_ is not obtained by simply adding F_IS_ and F_ST_: the intersection F_IS_. F_ST_ should be subtracted from this sum. The same result is obtained from the equation (1 − F_IT_) = (1 − F_IS_).(1 − F_ST_).

The probability of a male or female conceptus being homozygous by descent as to the alleles from any autosomal locus is the inbreeding coefficient F = 1/16, independently from the structure A, B, C, or D of the first-cousin marriage. The deleterious effects of pathological autosomal recessive alleles (occasionally carried in more than 1,000 different loci) has been easily demonstrated not only through empirical studies (that compare morbidity and mortality rates in the offspring of unrelated and consanguineous couples) but also by predictive theoretical models as well^4^.

The probabilities of a female concept being homozygous by descent as to alleles carried by an X-linked locus in subtypes A, B, C, and D are, respectively, F_A_ = 2(1/2)^5^ + (1/2)^3^ = 3/16, F_B_ = 2(1/2)^4^ = 1/8, F_C_ = 0, and F_D_ = 0; if the four subtypes occur in the population with approximately equal (1/4) frequencies, the average value of the inbreeding coefficient becomes F = 5/64 = 0.0781. The additional deleterious effect due to this should be not only modest but also very difficult to estimate because (1) the genes on the X chromosome represent just about five percent of all genes contained on the human genome; and (2) the deleterious alleles in the X loci are submitted to a selection process much stronger than the corresponding one to autosomes, because they are in hemizygous state among male individuals, and therefore with a much smaller frequency than their autosomal counterparts^5^.

Crow and Mange^6^ introduced a method that uses the frequencies of population surnames and marriages of persons with the same surname to estimate the different fixation indexes F_IT_, F_IS_ and F_ST_. The original method can be applied only to literate populations with a fixed manner of surname transmission and with no asymmetry in types A, B, C, and D. The method was revisited by several authors, including Cabello and Krieger^7^, who made room in it for the asymmetry of patrilineal subtype D.

The first (and main) objective of the present paper is to provide explanation on the frequency asymmetry of types A, B, C, and D, a topic still not solved in a completely satisfactory manner in the literature.

The second objective of this work is to adapt the isonomy method taking into account not only distortions in the frequencies of types A, B, C, and D but also the variable mode of surname transmission in poorly acculturated populations without fixed rules.

## Results

### Asymmetry of first-cousin subtypes A, B, C, and D

With the aim of obtaining a global vision of the distribution of subtypes A, B, C, and D, we reduced drastically the number of population samples shown in Tables IS to IVS of supplementary material, agglomerating all regional groups shown to be homogeneous after the application of standard chi-squared heterogeneity tests (results detailed on supplementary Table VS). The various samples from four countries resisted to this process of coalescence (Israel; Japan; Jordan; and Spain). The 18 groups from Japan could be coalesced in only two subpopulations, rural (14 groups) and urban (four groups). In the case of Spain, two groups (B-C) out of the four could be considered as one population. With the application of this procedure, the total of 126 different individual samples listed in Tables IS to IVS (supplementary material) reduced to just 28, as shown in Table 1, which summarizes the results of descriptive analysis from these new results.

**Table 1:** Descriptive analysis of the results obtained after agglutinating all geographically similar subsamples shown in Tables IS to IVS (supplementary material): (1) Austria, (2) Belgium (two agglutinated subsamples), (3) NE Brazil (Freire-Maia; four agglutinated subsamples), (4) S/SE Brazil (Freire-Maia; seven agglutinated subsamples), (5) NE Brasil (Paraíba; 35 agglutinated subsamples), (6) SE Brazil (Laboratório de Genética Humana USP; 32 agglutinated subsamples), (7) Chile, (8) England (three agglutinated subsamples), (9) Germany (three agglutinated subsamples), (10) India (three agglutinated subsamples), (11–14) Israel A, B, C and D, (15) Italy, (16) Japan (four agglutinated urban subsamples), (17) Japan (14 agglutinated rural subsamples), (18–20) Jordan A, B and C, (21) Korea, (22) Norway, (23) Pakistan, (24) Spain A, (25) Spain B + C (two agglutinated subsamples), (26) Spain D, (27) Sweden, (28) United States.

Sample	Locality	N	a = A/N	b = B/N	c = C/N	d = D/N	a + b	c + d	%fem	%mal	F
1	Austria	822	0.33333	0.27981	0.20560	0.18127	0.61314	0.38687	0.57604	0.42398	0.09748
2	Belgium	210	0.23333	0.26667	0.23333	0.26667	0.50000	0.50000	0.48333	0.51667	0.07708
3	Brazil NE old	1809	0.22886	0.24433	0.20122	0.32559	0.47319	0.52681	0.45164	0.54836	0.07345
4	Brazil SE old	1,036	0.26158	0.24807	0.22297	0.26737	0.50965	0.49034	0.49710	0.50289	0.08006
5	Brazil NE new	909	0.27063	0.20462	0.26183	0.26293	0.47525	0.52476	0.50385	0.49616	0.07632
6	Brazil SE new	989	0.31345	0.28514	0.21234	0.18908	0.59859	0.40142	0.56219	0.43782	0.09441
7	Chile	17	0.05882	0.35294	0.11765	0.47059	0.41176	0.58824	0.29412	0.70588	0.05515
8	England	296	0.34122	0.22973	0.20608	0.22297	0.57095	0.42905	0.55912	0.44088	0.09270
9	Germany	1617	0.29314	0.27149	0.21831	0.21707	0.56463	0.43538	0.53804	0.46197	0.08890
10	India	290	0.16552	0.30000	0.27586	0.25862	0.46552	0.53448	0.45345	0.54655	0.06854
11	Israel A	598	0.31104	0.21405	0.17726	0.29766	0.52509	0.47492	0.50670	0.49331	0.08508
12	Israel B	212	0.31132	0.16981	0.09434	0.42453	0.48113	0.51887	0.44340	0.55660	0.07960
13	Israel C	456	0.13816	0.24561	0.15351	0.46272	0.38377	0.61623	0.33772	0.66228	0.05661
14	Israel D	509	0.19843	0.15324	0.14538	0.50295	0.35167	0.64833	0.34774	0.65226	0.05636
15	Italy	4,384	0.27714	0.29037	0.20963	0.22286	0.56751	0.43249	0.52714	0.47286	0.08826
16	Japan URB	2,263	0.35307	0.26646	0.19620	0.18427	0.61953	0.38047	0.58440	0.41560	0.09951
17	Japan RUR	1648	0.25364	0.30825	0.22209	0.21602	0.56189	0.43811	0.51881	0.48119	0.08609
18	Jordan A	303	0.08911	0.09901	0.05611	0.75578	0.18812	0.81189	0.16667	0.83334	0.02908
19	Jordan B	360	0.15278	0.16667	0.07778	0.60278	0.31945	0.68056	0.27500	0.72500	0.04948
20	Jordan C	487	0.26694	0.18480	0.11499	0.43326	0.45174	0.54825	0.41684	0.58316	0.07315
21	Korea	54	0.35185	0.37037	0.12963	0.14815	0.72222	0.27778	0.60185	0.39815	0.11227
22	Norway	112	0.25000	0.22321	0.25000	0.27679	0.47321	0.52679	0.48660	0.51340	0.07478
23	Pakistan	516	0.25581	0.25194	0.17442	0.31783	0.50775	0.49225	0.46899	0.53101	0.07946
24	Spain A	3,160	0.31171	0.27152	0.21076	0.20601	0.58323	0.41677	0.55285	0.44715	0.09239
25	Spain B + C	1652	0.27966	0.27482	0.21489	0.23063	0.55448	0.44552	0.52452	0.47549	0.08679
26	Spain D	3,250	0.26185	0.27815	0.21846	0.24154	0.54000	0.46000	0.51016	0.48984	0.08387
27	Sweden	34	0.23529	0.44118	0.17647	0.14706	0.67647	0.32353	0.54412	0.45589	0.09926
28	United States	104	0.37500	0.19231	0.21154	0.22115	0.56731	0.43269	0.57692	0.42308	0.09435

N : sample size (number of first cousin couples); a, b, c, d, a + b, c + d : frequencies of subtypes A, B, C, D, A + B e C + D; %fem e %mal: percentages of women and men among the parental sibs of the first cousins; F: average inbreeding coefficient of the feminine offspring, taking into account the observed frequencies a, b, c, and d.

**Figure 2:**
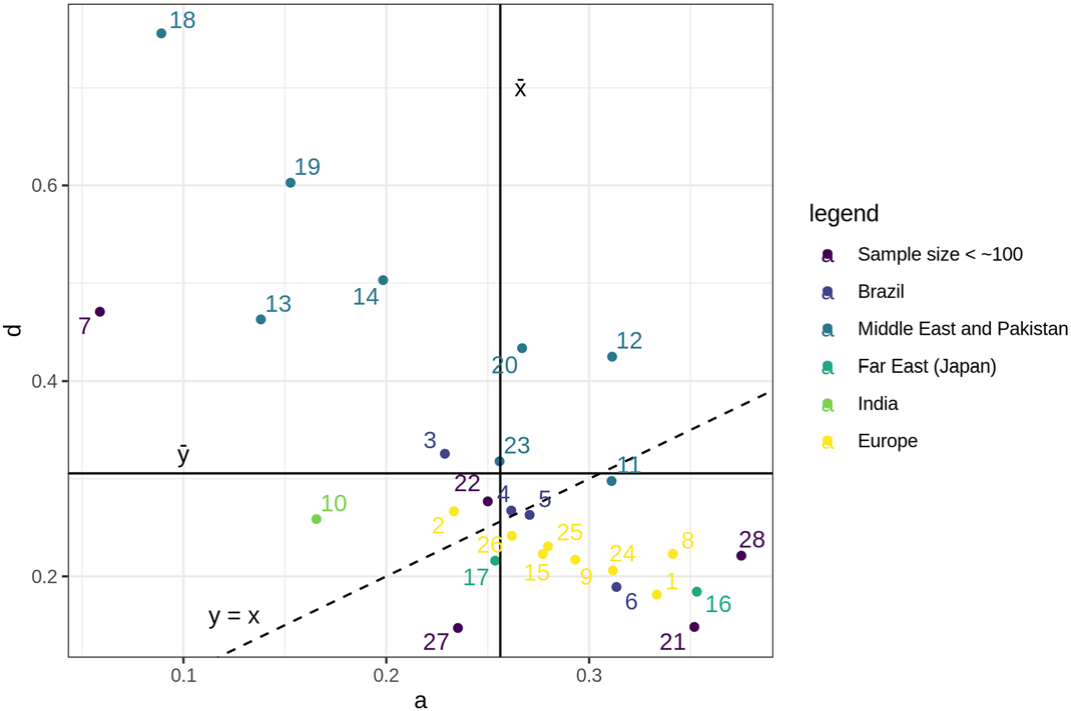
Distribution of a = A/N and d = D/N values in the samples described on Table 1, identified by the figures shown at the leftmost column of this table.

All data from Table 1 are plotted in the graph of Fig. 2, in which the abscissae axis (x) corresponds to the values of a = A/N and the ordinates axis (y) to the values of d = D/N. The graph shows also the straight lines that represent the average values of a and d estimated from all populations shown, as well as the line a = d. The plotted circles in purple correspond to samples with small sample sizes (less than 100): 6 Chile, 20 (Korea), 21 (Norway), 26 (Sweden) and 27 (United States). These numbers, as well all others shown in Fig. 2, correspond to the leftmost figures identifying the population samples listed on Table 1.

The influence of ruralization and urbanization and of other factors such as different mobility rates among males and females on the frequency of first-cousin marriage subtypes A, B, C, and D has been discussed in the literature^1,8^, however without any results that can be generalized, probably due to temporal, geographical and cultural differences in relation to the concepts of what is rural or urban in different populations. For example, a community considered as rural in present Belgium probably corresponds to urban life in a large city fairly developed in a poorly developed and industrialized country, in which an area categorized as rural in present times surely corresponds to a rural area in fully developed and industrialized countries many decades ago. Taking this reasoning into account, the agglomeration of rural or urban areas from geo-politically distinct regions makes no sense at all. The Japanese reports (as well as other researches developed in Japan by American geneticists), on the other hand, have an astonishing amount of reliable information as to the rural or urban character of the samples, but their data were used generally with the main aim of comparing the viability, morbidity, and mortality of the offspring in subtypes A, B, C, and D. The heterogeneity test in 14 samples collected in rural areas (Hirado A, Hoshino, Ina, Kurogi, Kyushu, Mishima, Nansei, Nanto, Okayama, Onodani, Oshima, Shizuoka A, Shizuoka B, Shizuoka C) showed that they could be coalesced, the same occurring with four urban localities (Fukuoka, Hirado B, Hiroshima A, Hiroshima B) (Table VS). Testing the hypothesis that the distribution of subtypes A, B, C, and D is aleatory (expected frequency of each class = 1/4) in the urban and rural samples resulting from four and 14 subsamples respectively, we found that the observed frequencies are significantly different from the expected by chance (p-value < 0.0001). By comparing both coalesced samples through a chi-squared test on a contingency table, a very significant chi-squared value (p-value < 0.0001) was obtained; the analysis of the adjusted residuals (Pearson/Haberman test) showed that there is a significant increase of subtype A and a significant decrease of the other subtypes (B, C, and D) in the urban communities and the opposite in rural ones.

**Figure 3:**
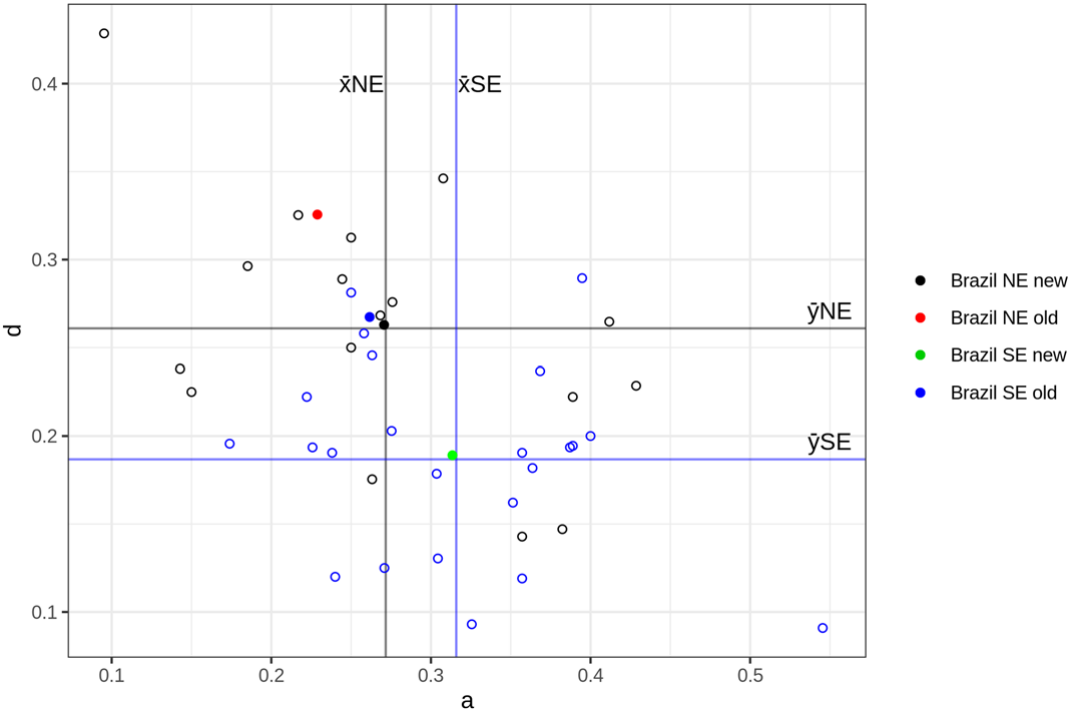
Distribution of a = A/N and d = D/N values in the Brazilian samples (see details and explanations on the text above).

Since the use of variables d = D/N and a = A/N, respectively on ordinates (y) and abscissas (x) axes in Fig. 2 seem to be sufficient for causing a reasonably good dispersion in the set of plotted data, we present in detail, on Fig. 3, the results corresponding just to the samples collected in Brazil. The individual values of the samples are represented by clear dots, with the exclusion of all subsamples with size less than 20. With this strategy the number of samples collected in the state of Paraiba in NE Brazil reduced from 35 to 17. The filled symbols n1, n2, s1, and s2 correspond to the average values of a and d in the samples of NE^1,2^, NE (present work), S/SE^1,2^, and SE (present work) regions. The graph shows also the average values of a and d for the NE and SE regions, that are situated exactly in the intersection of the straight lines corresponding to the average allover values of a and d for these regions.

Figure 3 shows that subtype D frequency value is predominant in the old NE sample while subtype A frequency value is predominant in the new SE sample, the first above the global averages of d for both NE and SE regions and below the global average of a for both regions; and the second value one below the global averages of d for both NE and SE regions and above the global average of a for both regions. The values corresponding to the most recent NE sample and to the old S/SE regions are very similar and exactly intermediary in relation to the global allover average values of a and b.

### Estimate of F_IT_ through the analysis of the pattern of surname transmission in Northeastern Brazil

The method of Crow and Mange^6^, briefly described in the lines below for the case of first cousin marriages, is based on the existence of: (1) rigid and fixed rules of surname transmission (generally or exclusively patrilineal or matrilineal); (2) a balanced (random) distribution of subtypes A, B, C, and D; and (3) surnames that have been generated just once in the population; under this last hypothesis, individuals with the same surname must have some degree of biological relationship.

If the observed population frequency of couples with the same surname is P, the approximate value of F_IT_ can be obtained from F_IT_ = P/4. If p_i_ and q_i_ are the proportions of men and women with a given surname, the population expected frequency of couples with this surname will be, on the hypothesis of random mating, p_i_q_i_, and its contribution to the inbreeding coefficient will be p_i_q_i_/4; and the contribution of all surnames to the random inbreeding coefficient will be F_ST_ = ∑p_i_q_i_/4. From F_IT_ = F_ST_ + F_IS_ − F_IS_F_ST_ we obtain the estimate of the non-random inbreeding coefficient F_IS_ = (F_IT_ − F_ST_)/(1 − F_ST_) = (P − ∑p_i_q_i_)/(4 − ∑p_i_q_i_) obtained from the analysis of surname population frequencies. Under the hypothesis that surnames occur with same frequencies among males and females (p_i_ = q_i_), it comes out that p_i_q_i_ = p_i_^2^; and the above formula can therefore be simplified to F_IS_ = (P − ∑p_i_^2^)/(4 − ∑p_i_^2^).

The authors of the method called attention to the problems of its application in poorly acculturated populations, without fixed rules of surname transmission. In the lines below we adapt the method to the population of the rural municipality of Brejo dos Santos, in the state of Paraiba in the Brazilian NE region, taking into account (1) the non-random distribution of sexes among the pairs of parental sibs of the consanguineous couples; and (2) the irregular mode of surname transmission in the population, that can take place: (1) strictly through the father; (2) strictly through the mother; (3) with an uncertain mode (case that takes place when both parents have the same surname that is transmitted to their offspring); and (4) when the offspring has surnames different from those of both parents.

#### Mode of surname transmission in the population

The studied sample was composed by 538 individuals with twice-checked information; in 243 of them the offspring surname was received only from the father, in 112 it was transmitted only by the mother; in 95 cases the surname didn’t originate from either parent; and in 88 cases the transmission mode could not be ascertained (parental pair with the same surname). The data from 90 women that had the marriage surname of their spouses, without reliable information as to their family surname, were previously excluded from the sample. Assuming that in the 88 cases of couples with the same surname the transmission took place patri- or matrilinearly after the proportions 243 : 112 respectively, we obtain the corrected proportions of transmission occurring patri- and matrilinearly, respectively, 243(1 + 88/355)/538 = 0.5636 and 112(1 + 88/355)/538 = 0.2598.

#### Surname distribution in the population and in the couples

In the calculations shown below, we considered 233 marriages that did not contain the 90 women without reliable information as to their family surname. Some men and some women from this population married twice or more times; this explains the fact that the totals of men and women that married are smaller than the total number of marriages (233), corresponding respectively to 219 and 225.

The masculine surnames were the following: Silva (63/219 = 0.288), Oliveira (24/219 = 0.110), Sousa (17/219 = 0.078), Costa (9/219 = 0.041), Diniz and Lima (8/219 = 0.037), Santos (7/219 = 0.032), Freitas and Melo (6/219 = 0.027), Bezerra (4/219 = 0.018), Almeida, Barbosa, Barreto, Dantas, Pereira, and Sobrinho (3/219 = 0.014); all other masculine surnames occurred each in just 1 or 2 individuals, therefore with a frequency of less than 1%.

The most common feminine surnames were: Silva (52/225 = 0.231), Conceição (23/225 = 0.102), Sousa (22/225 = 0.098), Freitas (19/225 = 0.084), Oliveira (17/225 = 0.076), Lima (9/225 = 0.040), Diniz (6/225 = 0.027), Araujo, Costa, Jesus, Sá, and Santos (4/225 = 0.018), Bezerra, Brito, Dantas, Guedes, Melo, and Vieira (3/225 = 0.013); all other surnames occurred in just 1 or 2 individuals each, therefore with a frequency of less than 1%.

Considering the total sample of 444 male and female individuals, the surnames that occurred in more than 4 individuals (therefore with a frequency larger than 1%) were: Silva (115/444 = 0.259), Oliveira (41/444 = 0.092), Sousa (39/444 = 0.088), Freitas (25/444 = 0.056), Conceição (23/444 = 0.052), Lima (17/444 = 0.038), Diniz (14/444 = 0.032), Costa (13/444 = 0.029), Santos (11/444 = 0.025), Melo (9/444 = 0.020), Bezerra (7/444 = 0.016), Araújo and Dantas (6/444 = 0.014), Barreto and Sá (5/444 = 0.011).

#### Estimation of fixation indexes F_IT_, F_ST_, and F_IS_

Using the data from Table VIS and applying to them, without any correction, the formulas proposed by Crow and Mange^6^, we obtain

$$ {\text{P }} = \, 39/233 \, = \, 0.1674 $$

$$ \Sigma {\text{p}}_{{\text{i}}} {\text{q}}_{{\text{i}}} = \, 0.0901 $$

$$ {\text{F}}_{{{\text{IT}}}} = {\text{P}}/4 = 0.0418 $$

$$ {\text{F}}_{{{\text{ST}}}} = \Sigma {\text{p}}_{{\text{i}}} {\text{q}}_{{\text{i}}} /4 = 0.0225 $$

$$ {\text{F}}_{{{\text{IS}}}} = \left( {{\text{F}}_{{{\text{IT}}}} - {\text{F}}_{{{\text{ST}}}} } \right)/\left( {1 - {\text{F}}_{{{\text{ST}}}} } \right) = 0.0197 $$

As shown in Table IS, in the municipality of Brejo dos Santos the frequencies of subtypes A, B, C and D of first cousin marriages were respectively 0.2169, 0.2530, 0.2048, and 0.3253. Taking into account that in this locality the frequencies with which the surname is patrilinearly and matrilinearly transmitted are 0.5636 and 0.2598 respectively, a gross correction of the factor 0.25 that occurs in the formulas of Crow and Mange can be obtained from 0.2169 × 0.2598 + 0.3253 × 0.5636 = 0.2397 ~ 0.24. The estimates of the three fixation indexes F_IT_, F_ST_, and F_IS_ then become

$$ {\text{F}}_{{{\text{IT}}}} = 0.24 \times {\text{P}} = 0.0402 $$

$$ {\text{F}}_{{{\text{ST}}}} = 0.24 \times \Sigma {\text{p}}_{{\text{i}}} {\text{q}}_{{\text{i}}} = 0.0216 $$

$$ {\text{F}}_{{{\text{IS}}}} = \left( {{\text{F}}_{{{\text{IT}}}} - {\text{F}}_{{{\text{ST}}}} } \right)/\left( {1 - {\text{F}}_{{{\text{ST}}}} } \right) = 0.0190 $$

#### Rates of intentional (inbreeding strictly speaking) and random consanguineous matings in NE Brazil

The importance of the surname method is that it enables the indirect estimation of the factors responsible for the consanguineous marriages. Since all figures obtained for the estimates F_IT_, F_ST_, and F_IS_ are positive, we can obtain the approximate proportion of first cousin marriages due to intentional factors (inbreeding), which is 0.47 or about 50%.

Table 2 (adapted from Weller et al.^9^) shows the estimates of population size, frequency of consanguineous marriages and the average value of the inbreeding coefficient F_IT_ of a set of rural localities in the state of Paraiba in NE Brazil. The table originally published contains data from 39 localities, not including the data from Gurjão and Lagoa Seca (shown in Table IS) and including six localities not shown in the same table (Belém, Cajazerinhas, Matinhas, Poço Dantas, Sousa and Vierópolis). Brejo dos Santos is included in Table 2 among the localities with a population size of less than 10,000.

**Table 2:** Estimated population sizes (n), observed frequencies of consanguineous mating (frcm), and average fixation index (F_IT_) in localities from NE Brazil (state of Paraíba).

Sample	n	frcc	F_IT_
1	28,299	0.1929	0.00651
2	33,031	0.1623	0.00380
3	55,469	0.1671	0.00509
4	13,821	0.1912	0.00724
5	4,416	0.2049	0.00604

All data were adopted and condensed from Weller et al.^9^, taking into account population size intervals of 10,000. (1) Average values of Catolé do Rocha and Pombal, (2) São Bento; (3) Sousa; (4) Uiraúna; (5) average values of all other localities (including Brejo dos Santos), each with a population size less than 10,000.

**Figure 4:**
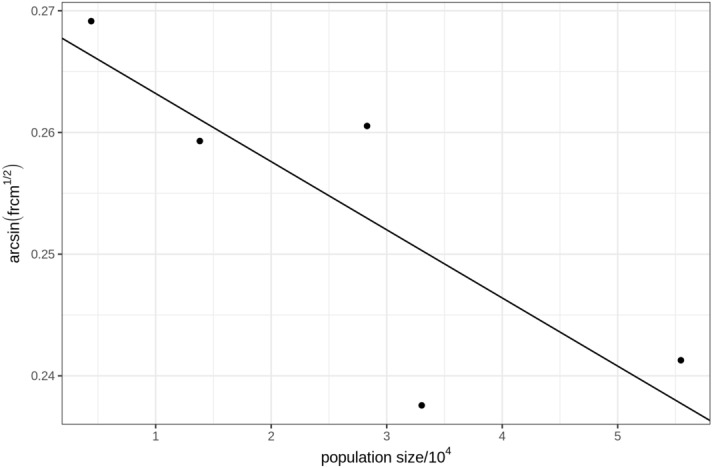
Indication of a negative correlation between the frequency of consanguineous mating and population sizes in the state of Paraiba in NE Brazil (data taken from Weller et al.^9^). The black line corresponds to the regression line of the data.

Since the surname method indicated a frequency of about 50% of consanguineous marriages taking place due to intentional factors (strict sense inbreeding), it is expected that an inverse correlation (or at least a tendency) exists between the population sizes and the frequency of consanguineous mating occurring in them. In order to verify this point, the data from Table 2 (frcm and n) were submitted to a simple model of linear regression analysis, after applying to the dependent variable (frcm) the transformation arc-sin(x)^1/2^ with the aim of normalizing its distribution. The results of this analysis revealed the existence of a tendency, with the values of frcm being roughly estimated by the formula frcm = 0.2688—0.0056n; r^2^ = 0.6675; F(1;3) = 6.021 (p-value = 0.09), as shown in the graph of Fig. 4.

## Discussion and conclusions

Consanguineous marriages still take place at significant proportions worldwide (at least 10% of all human unions), being a frequent practice not only among Muslim populations (in the Middle East, North Africa, and West Asia) and in some regions of India but also in many areas (especially rural, isolated, and underdeveloped ones) throughout the whole world^10–14^. By far the most common type of consanguineous marriage is the first-cousin one, which accounts generally for proportions as high as 30% to 50% or even more of all mating taking place between relatives in a given population^11,13,14^. Although there is currently no general consensus about the reasons that favor the occurrence of consanguineous mating in such high rates, and probably because of its very complicated multifaceted nature, the issue has been subjected to a large number of different well-planned studies, an important list of which is found in www.consang.net14. These studies were able to suggest explaining factors related to social, religious, cultural, political, and economical status, as well as the smaller size of isolated rural populations^13,15^. For example, in Arab populations consanguineous marriages are favoured because unions between non-relatives are thought to be less stable, since in marital disputes the husband's family would side with the consanguineous wife^11,16–18^.

An important issue is why particular types of first cousin unions are strongly favored in some major populations, e.g. patrilineal parallel cousin marriage (type D) in Muslim Arab communities, but prohibited in others^19^, a result also observed by us. The scatter diagram of Fig. 2 draws immediate attention to this fact. In more than half of the samples we selected (numbered as 12, 13, 17, 18, and 22), corresponding to Near and Middle East populations with more rigid and strict traditional patriarchal rules, the subtype D is larger than A. This tendency might be explained by the simple fact that in strongly patriarchally rooted populations or communities the father of the bride or bridegroom, when arranging a consanguineous marriage within his family, will preferentially choose the partner cousin among the offspring of his brothers in flagrant detriment to the offspring of his sisters.

This tendency is strikingly downplayed in the majority of other population groups, as the graph of Fig. 2 clearly shows. When Southern Indian Hindus are considered, for example, the situation is reversed, because parallel cousin unions (types A and D) are forbidden in Hindu society^20^. This simple fact probably explains per se the lower frequency (of about 40%) of types A and D in the Indian samples (123 out of 290 first-cousin marriages) we included in the present study.

The separate analysis of the four Brazilian samples (Table 1 and Fig. 3) indicates an overwhelmingly predominant increase of the subtype D in the old NE sample, above the overall average values of D for both geographical regions NE and SE and far below from the global averages value of A for the same regions. This predominance is fairly suggestive of a more stringent application of patriarchal rules, a common issue in old rural areas of NE Brazil; just the opposite is observed in relation to the average values of A for both regions. The whole situation is completely inverted in present SE Brazil (recent sample from the Laboratory of Human Genetics at USP), with increase of subtype A and corresponding decrease of subtype D. The global values corresponding to the population samples collected recently in the state of Paraíba (NE Brazil) and the old data from S/SE Brazil are very similar, in a position exactly intermediary to the overall global average values of A and D. This finding seems to be strongly correlated to the geopolitical transformations of NE and SE that took place in Brazil along the time.

In order to verify these facts globally, we performed a correlation analysis comparing all A and D percentage values from Table 1 with the corresponding 2017 HDI (human development index taken from the WWW site U. N. Development Programme) of all countries and regions listed there. We observed an almost significant (p-value = 0.0573) positive correlation (Spearman's rank correlation coefficient r = 0.3635) between HDI and the frequency of A subtype and a significant (p-value = 0.0122) negative correlation (Spearman's rank correlation coefficient r = − 0.4669) between HDI and the frequency of D subtype (Fig. 1S of supplementary material).

All these results are corroborated, at least partly, by the behavior of A and (B + C + D) subtypes in rural and urban Japanese communities.

The simple, rough method we proposed to correct the method of Crow and Mange^6^ for distribution asymmetries of the four subtypes of first-cousin marriages and the absence of fixed population rules of surname transmission provided an estimate of 0.2397 (estimated from the observed frequencies of subtypes A, B, C, D, and the corrected rates of paternal and maternal surname transmission in Brejo dos Santos) that is not significantly different from the factor 1/4 = 0.25 used in the original method. This was just coincidental and probably applicable just to that population aggregate. For other population samples with any asymmetry in the distribution of subtypes A, B, C, D and without very fixed rules of surname transmission, the method of Crow and Mange should always be corrected as proposed, despite the fact that our calculations did not take into account the complication of adopted surnames, a phenomenon that is fairly common in some countries.

The estimated frequency of F_IT_ in Brejo dos Santos, obtained from the genealogical analysis of the sampled population, was 0.00504^9^, a value significantly smaller than the one estimated by the surnames method. Some discrepancy is expected to occur because our estimate used only information from first-cousin marriages. As the estimated frequency of all types of consanguineous marriages in Brejo dos Santos is 0.1948, if all consanguineous marriages in the locality took place only among first cousins (coefficient of inbreeding 1/16 = 0.0625), the value of the total fixation index would be at least F_IT_ = 0.1948 . 0.0625 = 0.0122; then at least 1 – 0.00504/0.0122 = 0.5869 or about 60% of the consanguineous marriages occurring in the region takes place between relatives with a degree of biological relationship much smaller than the one prevailing between first cousins. A better, more plausible explanation for this discrepancy is the fact that the surname method estimates the degree of relationship even when a fraction of the interviewed individuals doesn’t know that they are actually married to relatives.

The real importance of the method, however, is that it enabled the indirect estimation of the relative frequencies of consanguineous marriages due to strictly inbreeding causes (intentional or *strictu* sensu inbreeding) and random inbreeding (caused by small population effective numbers or other aleatory phenomena). For the studied population group the estimated rate of inbreeding that is intentional was about 50%. Should this be true, there should exist an association (or at least some tendency) between the population sizes and their corresponding frequencies of consanguineous mating. Using data provided by our group in a paper published recently (based on practically the same set of populations studied in the state of Paraíba in NE Brazil), and using a simple model of regression analysis, we showed that the values of the frequencies of consanguineous mating (frcm) in NE Brazil can be roughly estimated by the corresponding sizes of the populations (n) in which they occur by the formula frcm = 0.2688—0.0056n; r^2^ = 0.6675; F(1;3) = 6.021 (p-value = 0.09). The statistical non-significance of the probability test value indicates just a tendency, that could be explained by the paucity of available coalesced data pairs that validated the regression analysis (just five pairs, as shown by the graph on Fig. 4).

## Subjects and methods

The present work was based on large data sets personally collected by us (1) on a routine basis from the identification archives of the genetic counseling service of the Human Genetics Laboratory (Department of Genetics, Institute of Biosciences, University of São Paulo, São Paulo, SE Brazil) from 1979 to 2010, totaling anonymoulsy collected information on 989 marriages between first cousins; and (2) in 35 municipalities from the state of Paraíba in NE Brazil, totaling 909 marriages between first cousins and collected recently^21^. We performed also a comprehensive review of reliable published data in a significant number of reports from the literature and comprehensively reanalyzed all this material. All these data have been double checked thoroughly.

All data were analyzed by standard statistical methods used in population genetics and described in basic college textbooks such as Weir^22^ and Zar^23^. For this analysis we prepared computer programs/scripts in R (Copyright 2018 The R Foundation for Statistical Computing) and Liberty Basic (Copyright 1992–2010 Shoptalk Systems) languages.

All data we used in the present paper, together with their descriptive analysis, are detailed in the supplementary tables IS, IIS, IIIS, and IVS. Tables IS and IIS contain the original material of the present paper. Table IIIS details 11 Brazilian samples from the literature, collected and studied by Freire-Maia^1^ and Freire-Maia and Freire-Maia^2^. Table IVS summarizes the descriptive data from 48 samples described in the literature, excluding those from Brazil: Orel^24^; Deraemaeker^25^; Freire-Maia and Freire-Maia^2^; Villanueva et al*.*^26^; Haldane and Moshinsky^27^; Shields and Slater^28^; Nixon and Slater^29^; Ludwig^30,31^; Zerbin-Rüdin^32^; Sanghvi et al.^33^; Goldschmidt et al.^34^; Zlotogora and Shalev^35^; Barrai et al.^36^; Sharkia et al.^37^; Hamamy et al.^38^; Kang and Cho^39^; Oedegard and Herlofsen^40^; Shami^41^; Calderón et al.^42^; Slatis, et al.^43^; Morton^8^; Schull^44^; Hook and Schull^45^; Fujiki et al.^46^; Komai and Tanaka^47^; Yanase et al.^48^; Böök^49^; Deraemaeker, 1961 (Personal communication to Freire-Maia and Freire-Maia^2^).

### Ethics approval and consent to participate

The data sampling protocol and the consent procedure were reviewed and approved by the State University of Paraiba Ethics committee (CAAE: 67,426,017.6.0000.5187) and State University of São Paulo Ethics committee. It was in accordance with the principles of Resolution 466/12 of the Brazilian National Health Council. All participants or their guardians received verbal and written explanations regarding the study procedures, and when they agreed, they signed the informed consent form and institutional declaration of approval.

## Supplementary information

Supplementary information.

## Data Availability

The data analysed during the current study are available from the corresponding author on reasonable request.
