# Supplementary material for: The structure of first-cousin marriages in Brazil
Source: Sci Rep. 2020 Sep 23;10:15573. doi: 10.1038/s41598-020-72366-z (PMC7511957; doi:10.1038/s41598-020-72366-z)
Supplement: Supplementary file 1 — Supplementary information. [file 41598_2020_72366_MOESM1_ESM.docx]

**The structure of first-cousin marriages in Brazil**

Paulo A. Otto^1^, Renan B. Lemes^1^, Allysson A. Farias^1^, Mathias Weller^2^, Shirley O. A. Lima^2^, Victor Alves Albino^2^, Yanna Marques^2^, Eliete Pardono^1^, Magnolia A. P. Bocangel^1^, Silvana Santos^2^ *

**1 Departamento de Genética e Biologia Evolutiva, Instituto de Biociências, Universidade de São Paulo, Rua do Matão 277, 05508-090 São Paulo SP, Brazil**

**2 Núcleo de Estudos em Genética e Educação, Universidade Estadual da Paraíba, Rua das Baraúnas, s/n, Campina Grande, Paraíba, Brazil**

Corresponding author:

Prof Dr Silvana Santos

Rua das Baraúnas, s/n - Prédio da Integração Acadêmica - sala 329

Universidade Estadual da Paraíba - Campus I - Bodocongó

Campina Grande - Paraíba

+55 (83) 3344-5306

e-mail: silvanaipe@gmail.com, [silvanasantosuepb@gmail.com](mailto:silvanasantosuepb@gmail.com)

**TABLE IS** - Descriptive analysis of the samples from the state of Paraíba in NE Brazil (data collected from 35 different localities, totaling 909 marriages between first degree cousins. N : sample size (number of first cousin couples); a, b, c, d, a+b, c+d : frequencies of subtypes A, B, C, D, A+B e C+D; %fem e %mal: percentages of women and men among the parental sibs of the first cousins; F: average inbreeding coefficient of the feminine offspring, taking into account the observed frequencies a, b, c, and d.

| Locality | N | a=A/N | b=B/N | c=C/N | d=D/N | a+b | c+d | %fem | %mal | F |
| --- | --- | --- | --- | --- | --- | --- | --- | --- | --- | --- |
| Aparecida | 14 | 0.35714 | 0.21429 | 0.21429 | 0.21429 | 0.57143 | 0.42858 | 0.57143 | 0.42858 | 0.09375 |
| Assunção | 10 | 0.30000 | 0.10000 | 0.40000 | 0.20000 | 0.40000 | 0.60000 | 0.55000 | 0.45000 | 0.06875 |
| Bernardino Batista | 16 | 0.31250 | 0.31250 | 0.12500 | 0.25000 | 0.62500 | 0.37500 | 0.53125 | 0.46875 | 0.09766 |
| Boa Vista | 34 | 0.41176 | 0.05882 | 0.26471 | 0.26471 | 0.47058 | 0.52942 | 0.57353 | 0.42648 | 0.08456 |
| Bom Jesus | 4 | 0.25000 | 0.25000 | 0.25000 | 0.25000 | 0.50000 | 0.50000 | 0.50000 | 0.50000 | 0.07812 |
| Brejo dos Santos | 83 | 0.21687 | 0.25301 | 0.20482 | 0.32530 | 0.46988 | 0.53012 | 0.44578 | 0.55422 | 0.07229 |
| Camalau | 32 | 0.25000 | 0.25000 | 0.18750 | 0.31250 | 0.50000 | 0.50000 | 0.46875 | 0.53125 | 0.07812 |
| Carrapateira | 28 | 0.35714 | 0.21429 | 0.28571 | 0.14286 | 0.57143 | 0.42857 | 0.60714 | 0.39286 | 0.09375 |
| Catolé do Rocha | 41 | 0.26829 | 0.21951 | 0.24390 | 0.26829 | 0.48780 | 0.51219 | 0.50000 | 0.50000 | 0.07774 |
| Caturite | 13 | 0.23077 | 0.00000 | 0.38462 | 0.38462 | 0.23077 | 0.76924 | 0.42308 | 0.57693 | 0.04327 |
| Emas | 9 | 0.33333 | 0.22222 | 0.22222 | 0.22222 | 0.55555 | 0.44444 | 0.55555 | 0.44444 | 0.09028 |
| Gado Bravo | 40 | 0.15000 | 0.27500 | 0.35000 | 0.22500 | 0.42500 | 0.57500 | 0.46250 | 0.53750 | 0.06250 |
| Gurjão | 15 | 0.40000 | 0.00000 | 0.20000 | 0.40000 | 0.40000 | 0.60000 | 0.50000 | 0.50000 | 0.07500 |
| Jericó | 45 | 0.24444 | 0.24444 | 0.22222 | 0.28889 | 0.48888 | 0.51111 | 0.47777 | 0.52222 | 0.07639 |
| Lagoa | 35 | 0.42857 | 0.17143 | 0.17143 | 0.22857 | 0.60000 | 0.40000 | 0.60000 | 0.40000 | 0.10179 |
| Lagoa Seca | 5 | 0.00000 | 0.60000 | 0.40000 | 0.00000 | 0.60000 | 0.40000 | 0.50000 | 0.50000 | 0.07500 |
| Mato Grosso | 15 | 0.13333 | 0.46667 | 0.26667 | 0.13333 | 0.60000 | 0.40000 | 0.50000 | 0.50000 | 0.08333 |
| Nova Floresta | 3 | 0.00000 | 0.33333 | 0.33333 | 0.33333 | 0.33333 | 0.66666 | 0.33333 | 0.66666 | 0.04167 |
| Parari | 13 | 0.30769 | 0.07692 | 0.38462 | 0.23077 | 0.38461 | 0.61539 | 0.53846 | 0.46154 | 0.06731 |
| Pombal | 78 | 0.30769 | 0.11538 | 0.23077 | 0.34615 | 0.42307 | 0.57692 | 0.48077 | 0.51923 | 0.07211 |
| Quixaba | 4 | 0.25000 | 0.00000 | 0.25000 | 0.50000 | 0.25000 | 0.75000 | 0.37500 | 0.62500 | 0.04688 |
| Santa Cruz | 42 | 0.14286 | 0.30952 | 0.30952 | 0.23810 | 0.45238 | 0.54762 | 0.45238 | 0.54762 | 0.06548 |
| Santa Helena | 36 | 0.38889 | 0.19444 | 0.19444 | 0.22222 | 0.58333 | 0.41666 | 0.58333 | 0.41666 | 0.09722 |
| Sant. dos Garrotes | 34 | 0.38235 | 0.23529 | 0.23529 | 0.14706 | 0.61764 | 0.38235 | 0.61764 | 0.38235 | 0.10110 |
| Santo André | 15 | 0.46667 | 0.33333 | 0.20000 | 0.00000 | 0.80000 | 0.20000 | 0.73334 | 0.26667 | 0.12917 |
| São Bentinho | 6 | 0.33333 | 0.50000 | 0.16667 | 0.00000 | 0.83333 | 0.16667 | 0.66666 | 0.33334 | 0.12500 |
| São Bento | 27 | 0.18519 | 0.11111 | 0.40741 | 0.29630 | 0.29630 | 0.70371 | 0.44445 | 0.55556 | 0.04861 |
| São Domingos | 11 | 0.27273 | 0.00000 | 0.36364 | 0.36364 | 0.27273 | 0.72728 | 0.45455 | 0.54546 | 0.05114 |
| S. Dom. do Cariri | 15 | 0.40000 | 0.20000 | 0.20000 | 0.20000 | 0.60000 | 0.40000 | 0.60000 | 0.40000 | 0.10000 |
| São Francisco | 29 | 0.27586 | 0.13793 | 0.31034 | 0.27586 | 0.41379 | 0.58620 | 0.50000 | 0.50000 | 0.06896 |
| S. José B. da Cruz | 42 | 0.09524 | 0.11905 | 0.35714 | 0.42857 | 0.21429 | 0.78571 | 0.33334 | 0.66666 | 0.03274 |
| S. Seb. Umbuzeiro | 12 | 0.16667 | 0.00000 | 0.41667 | 0.41667 | 0.16667 | 0.83334 | 0.37500 | 0.62500 | 0.03125 |
| Tenório | 44 | 0.25000 | 0.25000 | 0.25000 | 0.25000 | 0.50000 | 0.50000 | 0.50000 | 0.50000 | 0.07812 |
| Uirauna | 57 | 0.26316 | 0.28070 | 0.28070 | 0.17544 | 0.54386 | 0.45614 | 0.54386 | 0.45614 | 0.08443 |
| Zabele | 2 | 0.00000 | 0.50000 | 0.50000 | 0.00000 | 0.50000 | 0.50000 | 0.50000 | 0.50000 | 0.06250 |

**TABLE IIS** - Descriptive analysis of the sample of couples of first cousins attending the genetic counselling service of the Laboratory of Human Genetics at USP in 32 consecutive years, from 1972 to 2010. N : sample size (number of first cousin couples); a, b, c, d, a+b, c+d : frequencies of subtypes A, B, C, D, A+B e C+D; %fem e %mal: percentages of women and men among the parental sibs of the first cousins; F: average inbreeding coefficient of the feminine offspring, taking into account the observed frequencies a, b, c, and d.

| year | N | a=A/N | b=B/N | c=C/N | d=D/N | a+b | c+d | %fem | %mal | F |
| --- | --- | --- | --- | --- | --- | --- | --- | --- | --- | --- |
| 1979 | 15 | 0.33333 | 0.33333 | 0.06667 | 0.26667 | 0.66666 | 0.33334 | 0.53333 | 0.46667 | 0.10417 |
| 1980 | 46 | 0.17391 | 0.43478 | 0.19565 | 0.19565 | 0.60869 | 0.39130 | 0.48912 | 0.51086 | 0.08696 |
| 1981 | 57 | 0.26316 | 0.24561 | 0.24561 | 0.24561 | 0.50877 | 0.49122 | 0.50877 | 0.49122 | 0.08004 |
| 1982 | 38 | 0.39474 | 0.23684 | 0.07895 | 0.28947 | 0.63158 | 0.36842 | 0.55264 | 0.44736 | 0.10362 |
| 1983 | 36 | 0.38889 | 0.25000 | 0.16667 | 0.19444 | 0.63889 | 0.36111 | 0.59722 | 0.40278 | 0.10417 |
| 1984 | 69 | 0.27536 | 0.33333 | 0.18841 | 0.20290 | 0.60869 | 0.39131 | 0.53623 | 0.46377 | 0.09330 |
| 1985 | 42 | 0.35714 | 0.28571 | 0.23810 | 0.11905 | 0.64285 | 0.35715 | 0.61905 | 0.38096 | 0.10268 |
| 1986 | 38 | 0.36842 | 0.26316 | 0.13158 | 0.23684 | 0.63158 | 0.36842 | 0.56579 | 0.43421 | 0.10197 |
| 1987 | 43 | 0.32558 | 0.30233 | 0.27907 | 0.09302 | 0.62791 | 0.37209 | 0.61628 | 0.38372 | 0.09884 |
| 1988 | 56 | 0.30357 | 0.21429 | 0.30357 | 0.17857 | 0.51786 | 0.48214 | 0.56250 | 0.43750 | 0.08371 |
| 1989 | 31 | 0.25806 | 0.35484 | 0.12903 | 0.25806 | 0.61290 | 0.38709 | 0.50000 | 0.50000 | 0.09274 |
| 1990 | 42 | 0.35714 | 0.19048 | 0.26190 | 0.19048 | 0.54762 | 0.45238 | 0.58333 | 0.41667 | 0.09077 |
| 1991 | 31 | 0.22581 | 0.38710 | 0.19355 | 0.19355 | 0.61291 | 0.38710 | 0.51614 | 0.48388 | 0.09073 |
| 1992 | 48 | 0.27083 | 0.31250 | 0.29167 | 0.12500 | 0.58333 | 0.41667 | 0.57292 | 0.42708 | 0.08984 |
| 1993 | 37 | 0.35135 | 0.35135 | 0.13514 | 0.16216 | 0.70270 | 0.29730 | 0.59460 | 0.40540 | 0.10980 |
| 1994 | 22 | 0.54545 | 0.13636 | 0.22727 | 0.09091 | 0.68181 | 0.31818 | 0.72726 | 0.27272 | 0.11932 |
| 1995 | 42 | 0.23810 | 0.23810 | 0.33333 | 0.19048 | 0.47620 | 0.52381 | 0.52382 | 0.47620 | 0.07441 |
| 1996 | 32 | 0.25000 | 0.25000 | 0.21875 | 0.28125 | 0.50000 | 0.50000 | 0.48438 | 0.51562 | 0.07812 |
| 1997 | 23 | 0.30435 | 0.39130 | 0.17391 | 0.13043 | 0.69565 | 0.30434 | 0.58696 | 0.41304 | 0.10598 |
| 1998 | 31 | 0.38710 | 0.19355 | 0.22581 | 0.19355 | 0.58065 | 0.41936 | 0.59678 | 0.40323 | 0.09678 |
| 1999 | 36 | 0.22222 | 0.30556 | 0.25000 | 0.22222 | 0.52778 | 0.47222 | 0.50000 | 0.50000 | 0.07986 |
| 2000 | 25 | 0.24000 | 0.28000 | 0.36000 | 0.12000 | 0.52000 | 0.48000 | 0.56000 | 0.44000 | 0.08000 |
| 2001 | 25 | 0.40000 | 0.24000 | 0.16000 | 0.20000 | 0.64000 | 0.36000 | 0.60000 | 0.40000 | 0.10500 |
| 2002 | 19 | 0.31579 | 0.36842 | 0.15789 | 0.15789 | 0.68421 | 0.31578 | 0.57895 | 0.42104 | 0.10526 |
| 2003 | 19 | 0.36842 | 0.26316 | 0.15789 | 0.21053 | 0.63158 | 0.36842 | 0.57895 | 0.42106 | 0.10197 |
| 2004 | 17 | 0.11765 | 0.47059 | 0.17647 | 0.23529 | 0.58824 | 0.41176 | 0.44118 | 0.55882 | 0.08088 |
| 2005 | 22 | 0.36364 | 0.18182 | 0.27273 | 0.18182 | 0.54546 | 0.45455 | 0.59092 | 0.40910 | 0.09091 |
| 2006 | 18 | 0.66667 | 0.16667 | 0.11111 | 0.05556 | 0.83334 | 0.16667 | 0.80556 | 0.19445 | 0.14583 |
| 2007 | 6 | 0.50000 | 0.50000 | 0.00000 | 0.00000 | 1.00000 | 0.00000 | 0.75000 | 0.25000 | 0.15625 |
| 2008 | 5 | 0.60000 | 0.40000 | 0.00000 | 0.00000 | 1.00000 | 0.00000 | 0.80000 | 0.20000 | 0.16250 |
| 2009 | 12 | 0.25000 | 0.16667 | 0.16667 | 0.41667 | 0.41667 | 0.58334 | 0.41667 | 0.58334 | 0.06771 |
| 2010 | 6 | 0.16667 | 0.33333 | 0.33333 | 0.16667 | 0.50000 | 0.50000 | 0.50000 | 0.50000 | 0.07292 |

**TABLE IIIS** - Descriptive analysis of other Brazilian samples from the literature, collected and studied in the fifties and sixties by Freire-Maia, 1958 and Freire-Maia and Freire-Maia, 1961. N : sample size (number of first cousin couples); a, b, c, d, a+b, c+d : frequencies of subtypes A, B, C, D, A+B e C+D; %fem e %mal: percentages of women and men among the parental sibs of the first cousins; F: average inbreeding coefficient of the feminine offspring, taking into account the observed frequencies a, b, c, and d.

| **Locality** | **N** | **a=A/N** | **b=B/N** | **c=C/N** | **d=D/N** | **a+b** | **c+d** | **%fem** | **%mal** | **F** |
| --- | --- | --- | --- | --- | --- | --- | --- | --- | --- | --- |
| **Belo_Horizonte_A** | 73 | 0.35616 | 0.19178 | 0.20548 | 0.24658 | 0.54794 | 0.45206 | 0.55479 | 0.44521 | 0.09075 |
| **Belo_Horizonte_B** | 327 | 0.23547 | 0.24465 | 0.24465 | 0.27523 | 0.48012 | 0.51988 | 0.48012 | 0.51988 | 0.07473 |
| **Barra** | 69 | 0.21739 | 0.27536 | 0.24638 | 0.26087 | 0.49275 | 0.50725 | 0.47826 | 0.52174 | 0.07518 |
| **Campanha** | 90 | 0.27778 | 0.27778 | 0.15556 | 0.28889 | 0.55556 | 0.44445 | 0.49445 | 0.50556 | 0.08681 |
| **Curitiba** | 113 | 0.18584 | 0.27434 | 0.25664 | 0.28319 | 0.46018 | 0.53983 | 0.45133 | 0.54868 | 0.06914 |
| **Pesqueira** | 560 | 0.24643 | 0.24286 | 0.17679 | 0.33393 | 0.48929 | 0.51072 | 0.45626 | 0.54376 | 0.07656 |
| **Petrolina** | 434 | 0.21659 | 0.23041 | 0.23963 | 0.31336 | 0.44700 | 0.55299 | 0.45161 | 0.54838 | 0.06941 |
| **São_Paulo_A** | 308 | 0.29870 | 0.22727 | 0.21753 | 0.25649 | 0.52597 | 0.47402 | 0.52110 | 0.47889 | 0.08442 |
| **São_Paulo_B** | 50 | 0.26000 | 0.32000 | 0.16000 | 0.26000 | 0.58000 | 0.42000 | 0.50000 | 0.50000 | 0.08875 |
| **Salvador** | 746 | 0.22386 | 0.25067 | 0.19303 | 0.33244 | 0.47453 | 0.52547 | 0.44571 | 0.55429 | 0.07331 |
| **Vitória** | 75 | 0.22667 | 0.28000 | 0.24000 | 0.25333 | 0.50667 | 0.49333 | 0.48667 | 0.51333 | 0.07750 |

**TABLE IVS** - Descriptive analysis of samples from the literature, excluding those from Brazil: Austria (Orel, 1932); Belgium A e B (Deraemaeker, 1958, and personal communication to Freire-Maia and Freire-Maia, 1961); Chile (Villanueva et al., 2014); England A (Haldane and Moshinsky, 1939), England B (Shields and Slater, 1956), England C (Nixon and Slater, 1957); Fukuoka, Japan; Germany A and B (Ludwig, 1948/49), Germany C (Zerbin-Rüdin, 1960); Hiroshima A, Japan; Hiroshima B, Japan; Hoshino, Japan; Ina, Japan; India A, B and C (Sanghvi, Varde and Master, 1956); Israel A and B (Goldschmidt et al., 1960); Israel C (Zlotogora and Shalev, 2014); Israel D (Sharkia et al., 2015); Italy (Barrai et al., 1962); Hirado A and B, Japan; Jordan A, B and C (Hamamy et al., 2005); Korea (Kang and Cho, 1959); Kurogi, Japan; Kyushu, Japan; Mishima, Japan; Nansei, Japan; Nanto, Japan; Norway (Oedegard and Herlofsen, 1957); Okayama, Japan; Onodani, Japan; Oshima, Japan; Pakistan (Shami, 1980); Shizuoka C, A, and B, Japan; Spain A, B, C, and D (Calderón et al., 2008); Sweden (Böök, 1957); United States (Slatis, Reis and Hoene, 1958). Data from samples studied in Japan, identified by the letter (J): Morton (1955), Tanaka (1955), Schull (1958), Hook and Schull (1973), Fujiki et al. (1968), Komai and Tanaka (1972), Yanase et al. (1973). N : sample size (number of first cousin couples); a, b, c, d, a+b, c+d : frequencies of subtypes A, B, C, D, A+B e C+D; %fem e %mal: percentages of women and men among the parental sibs of the first cousins; F: average inbreeding coefficient of the feminine offspring, taking into account the observed frequencies a, b, c, and d.

| Locality | N | a=A/N | b=B/N | c=C/N | d=D/N | a+b | c+d | %fem | %mal | F |
| --- | --- | --- | --- | --- | --- | --- | --- | --- | --- | --- |
| Austria | 822 | 0.33333 | 0.27981 | 0.20560 | 0.18127 | 0.61314 | 0.38687 | 0.57604 | 0.42398 | 0.09748 |
| Belgium A | 76 | 0.28947 | 0.28947 | 0.22368 | 0.19737 | 0.57894 | 0.42105 | 0.54604 | 0.45394 | 0.09046 |
| Belgium B | 134 | 0.20149 | 0.25373 | 0.23881 | 0.30597 | 0.45522 | 0.54478 | 0.44776 | 0.55224 | 0.06950 |
| Chile | 17 | 0.05882 | 0.35294 | 0.11765 | 0.47059 | 0.41176 | 0.58824 | 0.29412 | 0.70588 | 0.05515 |
| England A | 96 | 0.31250 | 0.21875 | 0.25000 | 0.21875 | 0.53125 | 0.46875 | 0.54688 | 0.45312 | 0.08594 |
| England B | 75 | 0.42667 | 0.18667 | 0.22667 | 0.16000 | 0.61334 | 0.38667 | 0.63334 | 0.36667 | 0.10333 |
| England C | 125 | 0.31200 | 0.26400 | 0.16000 | 0.26400 | 0.57600 | 0.42400 | 0.52400 | 0.47600 | 0.09150 |
| Fukuoka (J) | 1345 | 0.37100 | 0.26394 | 0.20297 | 0.16208 | 0.63494 | 0.36505 | 0.60446 | 0.39554 | 0.10256 |
| Germany A | 718 | 0.29109 | 0.28412 | 0.21588 | 0.20891 | 0.57521 | 0.42479 | 0.54109 | 0.45891 | 0.09009 |
| Germany B | 609 | 0.28407 | 0.25780 | 0.24302 | 0.21511 | 0.54187 | 0.45813 | 0.53448 | 0.46552 | 0.08549 |
| Germany C | 290 | 0.31724 | 0.26897 | 0.17241 | 0.24138 | 0.58621 | 0.41379 | 0.53793 | 0.46207 | 0.09310 |
| Hiroshima A (J) | 689 | 0.33237 | 0.26705 | 0.18433 | 0.21626 | 0.59942 | 0.40059 | 0.55806 | 0.44195 | 0.09570 |
| Hiroshima B (J) | 190 | 0.30000 | 0.28947 | 0.21579 | 0.19474 | 0.58947 | 0.41053 | 0.55263 | 0.44737 | 0.09243 |
| Hoshino (J) | 206 | 0.20388 | 0.32524 | 0.19903 | 0.27184 | 0.52912 | 0.47087 | 0.46602 | 0.53398 | 0.07888 |
| Ina (J) | 63 | 0.26984 | 0.31746 | 0.23810 | 0.17460 | 0.58730 | 0.41270 | 0.54762 | 0.45238 | 0.09028 |
| India A | 204 | 0.13725 | 0.32353 | 0.25000 | 0.28922 | 0.46078 | 0.53922 | 0.42402 | 0.57598 | 0.06618 |
| India B | 78 | 0.19231 | 0.25641 | 0.35897 | 0.19231 | 0.44872 | 0.55128 | 0.50000 | 0.50000 | 0.06811 |
| India C | 8 | 0.62500 | 0.12500 | 0.12500 | 0.12500 | 0.75000 | 0.25000 | 0.75000 | 0.25000 | 0.13281 |
| Israel A | 598 | 0.31104 | 0.21405 | 0.17726 | 0.29766 | 0.52509 | 0.47492 | 0.50670 | 0.49331 | 0.08508 |
| Israel B | 212 | 0.31132 | 0.16981 | 0.09434 | 0.42453 | 0.48113 | 0.51887 | 0.44340 | 0.55660 | 0.07960 |
| Israel C | 456 | 0.13816 | 0.24561 | 0.15351 | 0.46272 | 0.38377 | 0.61623 | 0.33772 | 0.66228 | 0.05661 |
| Israel D | 509 | 0.19843 | 0.15324 | 0.14538 | 0.50295 | 0.35167 | 0.64833 | 0.34774 | 0.65226 | 0.05636 |
| Italy | 4384 | 0.27714 | 0.29037 | 0.20963 | 0.22286 | 0.56751 | 0.43249 | 0.52714 | 0.47286 | 0.08826 |
| Hirado A | 526 | 0.23384 | 0.30798 | 0.24144 | 0.21673 | 0.54182 | 0.45817 | 0.50855 | 0.49144 | 0.08234 |
| Hirado B | 39 | 0.35897 | 0.23077 | 0.07692 | 0.33333 | 0.58974 | 0.41025 | 0.51282 | 0.48718 | 0.09615 |
| Jordan A | 303 | 0.08911 | 0.09901 | 0.05611 | 0.75578 | 0.18812 | 0.81189 | 0.16667 | 0.83334 | 0.02908 |
| Jordan B | 360 | 0.15278 | 0.16667 | 0.07778 | 0.60278 | 0.31945 | 0.68056 | 0.27500 | 0.72500 | 0.04948 |
| Jordan C | 487 | 0.26694 | 0.18480 | 0.11499 | 0.43326 | 0.45174 | 0.54825 | 0.41684 | 0.58316 | 0.07315 |
| Korea | 54 | 0.35185 | 0.37037 | 0.12963 | 0.14815 | 0.72222 | 0.27778 | 0.60185 | 0.39815 | 0.11227 |
| Kurogi (J) | 72 | 0.25000 | 0.20833 | 0.25000 | 0.29167 | 0.45833 | 0.54167 | 0.47916 | 0.52083 | 0.07292 |
| Kyushu (J) | 116 | 0.31034 | 0.28448 | 0.18966 | 0.21552 | 0.59482 | 0.40518 | 0.54741 | 0.45259 | 0.09375 |
| Mishima (J) | 76 | 0.15789 | 0.39474 | 0.15789 | 0.28947 | 0.55263 | 0.44736 | 0.43420 | 0.56578 | 0.07895 |
| Nansei (J) | 68 | 0.22059 | 0.32353 | 0.30882 | 0.14706 | 0.54412 | 0.45588 | 0.53676 | 0.46324 | 0.08180 |
| Nanto (J) | 46 | 0.21739 | 0.34783 | 0.21739 | 0.21739 | 0.56522 | 0.43478 | 0.50000 | 0.50000 | 0.08424 |
| Norway | 112 | 0.25000 | 0.22321 | 0.25000 | 0.27679 | 0.47321 | 0.52679 | 0.48660 | 0.51340 | 0.07478 |
| Okayama (J) | 129 | 0.33333 | 0.26357 | 0.24031 | 0.16279 | 0.59690 | 0.40310 | 0.58527 | 0.41473 | 0.09545 |
| Onodani (J) | 67 | 0.22388 | 0.23881 | 0.25373 | 0.28358 | 0.46269 | 0.53731 | 0.47015 | 0.52985 | 0.07183 |
| Oshima (J) | 40 | 0.15000 | 0.32500 | 0.37500 | 0.15000 | 0.47500 | 0.52500 | 0.50000 | 0.50000 | 0.06875 |
| Pakistan | 516 | 0.25581 | 0.25194 | 0.17442 | 0.31783 | 0.50775 | 0.49225 | 0.46899 | 0.53101 | 0.07946 |
| Shizuoka C (J) | 144 | 0.34722 | 0.29861 | 0.16667 | 0.18750 | 0.64583 | 0.35417 | 0.57986 | 0.42014 | 0.10243 |
| Shizuoka A (J) | 34 | 0.29412 | 0.41176 | 0.14706 | 0.14706 | 0.70588 | 0.29412 | 0.57353 | 0.42647 | 0.10662 |
| Shizuoka B (J) | 61 | 0.34426 | 0.37705 | 0.13115 | 0.14754 | 0.72131 | 0.27869 | 0.59836 | 0.40164 | 0.11168 |
| Spain A | 3160 | 0.31171 | 0.27152 | 0.21076 | 0.20601 | 0.58323 | 0.41677 | 0.55285 | 0.44715 | 0.09239 |
| Spain B | 1170 | 0.28632 | 0.27094 | 0.21368 | 0.22906 | 0.55726 | 0.44274 | 0.52863 | 0.47137 | 0.08755 |
| Spain C | 482 | 0.26349 | 0.28423 | 0.21784 | 0.23444 | 0.54772 | 0.45228 | 0.51452 | 0.48548 | 0.08493 |
| Spain D | 3250 | 0.26185 | 0.27815 | 0.21846 | 0.24154 | 0.54000 | 0.46000 | 0.51016 | 0.48984 | 0.08387 |
| Sweden | 34 | 0.23529 | 0.44118 | 0.17647 | 0.14706 | 0.67647 | 0.32353 | 0.54412 | 0.45589 | 0.09926 |
| United States | 104 | 0.37500 | 0.19231 | 0.21154 | 0.22115 | 0.56731 | 0.43269 | 0.57692 | 0.42308 | 0.09435 |

**Table VS**: Summary of chi-squared heterogeneity tests. NS: Number of different samples (subpopulations within a region or country population); d.f.: degrees of freedom. SCS: total of chi-squared tests. CSS: chi-squared of total data. HCS: heterogeneity chi-squared test. Probability test values in **bold** correspond to subpopulations that resisted to the process of agglutination.

| **Population** | **NS** | **SCS** | | | **CSS** | | | **HCS** | | |
| --- | --- | --- | --- | --- | --- | --- | --- | --- | --- | --- |
|  |  | value | d.f. | p-value | value | d.f. | p-value | value | d.f. | p-value |
| BRA-LHG | 32 | 134.8448 | 96 | 0.0055 | 41.1031 | 3 | <0.0001 | 93.7417 | 93 | 0.4589 |
| BRA-PB | 35 | 125,8011 | 105 | 0.0814 | 10.1587 | 3 | 0.0173 | 115.6504 | 102 | 0.1679 |
| BRA-NE | 4 | 70.2016 | 12 | <0.0001 | 62.0381 | 3 | 0.0001 | 8.1635 | 9 | 0.5170 |
| BRA-S/SE | 7 | 21.0194 | 21 | 0.4578 | 4.8494 | 3 | 0.1832 | 16.1700 | 18 | 0.5807 |
| Belgium | 2 | 5.0149 | 6 | 0.5419 | 0.9333 | 3 | 0.8174 | 4.0816 | 3 | 0.2528 |
| England | 3 | 21.5780 | 9 | 0.0103 | 13.4865 | 3 | 0.0037 | 8.0915 | 6 | 0.2315 |
| Germany | 3 | 35.1762 | 9 | 0.0001 | 28.5337 | 3 | <0.0001 | 6.6425 | 6 | 0.3552 |
| India | 3 | 27.8341 | 9 | 0.0010 | 12.0414 | 3 | 0.0072 | 15.7927 | 6 | 0.0149 |
| Israel | 4 | 384.5084 | 12 | <0.0001 | 279.0777 | 3 | <0.0001 | 105.4307 | 9 | **<0.0001** |
| Japan | 18 | 275.5694 | 54 | <0.0001 | 148.3598 | 3 | <0.0001 | 127.2096 | 51 | **<0.0001** |
| Japan rural | 14 | 93.7822 | 42 | <0.0001 | 35.2039 | 3 | <0.0001 | 58.5783 | 39 | 0.0228 |
| Japan urban | 4 | 181.7872 | 12 | <0.0001 | 163.9289 | 3 | <0.0001 | 17.8583 | 9 | 0.0369 |
| Jordan | 3 | 769.9160 | 9 | <0.0001 | 655.8922 | 3 | <0.0001 | 114.0238 | 6 | **<0.0001** |
| Spain | 4 | 145.4217 | 12 | <0.0001 | 119.2503 | 3 | <0.0001 | 26.1714 | 9 | **0.0019** |
| Spain B-C | 2 | 21.5252 | 6 | 0.0015 | 20.5085 | 3 | 0.0001 | 1.0167 | 3 | 0.7972 |

BRA-LGH, Laboratory of Human Genetics USP in SE Brazil; BRA-PB, Paraiba state in NE Brazil; BRA-NE, populations from the cities of Barra, Pesqueira, Petrolina, and Salvador in NE Brazil; BRA-S/SE, populations from the cities of Belo Horizonte (A and B), Campanha, Curitiba, São Paulo (A and B), Vitória in S/SE Brazil; Spain B-C, populations B and C from Spain; Japan, union of Japan groups 1 and 2; Japan group 1 (Hirado A, Hoshino, Ina, Kurogi, Kyushu, Mishima, Nansei, Nanto, Okayama, Onodani, Oshima, Shizuoka A, Shizuoka B, Shizuoka C); Japan group 2 (Fukuoka, Hirado B, Hiroshima A, Hiroshima).

**TABLE VIS** – Absolute observed frequencies of surnames in the population of Brejo dos Santos (state of Paraíba in NE Brazil). Column A: absolute frequencies of couples with the same surname; column B: absolute frequencies of masculine surnames; column C: absolute frequencies of feminine surnames. Totals: column A: 39/233 couples with the same surname; column B: 219 masculine surnames; coluna C: 225 feminine surnames.

| SURNAME | A | B | C | SURNAME | A | B | C | SURNAME | A | B | C |
| --- | --- | --- | --- | --- | --- | --- | --- | --- | --- | --- | --- |
| **Alexandre** | 0 | 1 | 0 | **Elias** | 0 | 1 | 0 | **Miranda** | 0 | 0 | 1 |
| **Almeida** | 0 | 3 | 1 | **Ferreira** | 0 | 2 | 0 | **Nascimento** | 0 | 0 | 2 |
| **Alves** | 0 | 1 | 2 | **Fidelis** | 0 | 1 | 0 | **Nogueira** | 0 | 0 | 1 |
| **Ambrosio** | 0 | 1 | 0 | **Figueiredo** | 0 | 0 | 1 | **Oliveira** | 3 | 24 | 17 |
| **Ambrozio** | 0 | 1 | 0 | **Franca** | 0 | 0 | 1 | **Oseas** | 0 | 1 | 0 |
| **Andrade** | 0 | 2 | 2 | **Francisco** | 0 | 0 | 1 | **Paiva** | 1 | 1 | 1 |
| **Araujo** | 0 | 2 | 4 | **Freitas** | 2 | 6 | 19 | **Paixao** | 0 | 1 | 0 |
| **Baltazar** | 0 | 1 | 1 | **Gomes** | 0 | 1 | 1 | **Pereira** | 0 | 3 | 0 |
| **Barbosa** | 0 | 3 | 1 | **Guedes** | 1 | 1 | 3 | **Pinheiro** | 0 | 1 | 0 |
| **Barreto** | 0 | 3 | 2 | **Guilhermino** | 0 | 1 | 0 | **Primo** | 0 | 1 | 0 |
| **Batista** | 0 | 1 | 0 | **Jesus** | 0 | 0 | 4 | **Remedios** | 0 | 0 | 1 |
| **Baudui** | 0 | 0 | 1 | **Jose** | 0 | 1 | 0 | **Rocha** | 0 | 0 | 1 |
| **Bezerra** | 0 | 4 | 3 | **Leandro** | 0 | 1 | 0 | **Rodrigues** | 0 | 0 | 1 |
| **Brito** | 0 | 0 | 3 | **Lima** | 1 | 8 | 9 | **As** | 0 | 1 | 4 |
| **Campos** | 0 | 1 | 0 | **Limeira** | 0 | 0 | 1 | **Salviano** | 0 | 1 | 0 |
| **Cardoso** | 0 | 2 | 0 | **Linhares** | 0 | 0 | 1 | **Santos** | 2 | 7 | 4 |
| **Carmo** | 0 | 0 | 1 | **Lins** | 0 | 2 | 0 | **Saturnino** | 0 | 1 | 0 |
| **Carneiro** | 0 | 0 | 1 | **Lourdes** | 0 | 0 | 2 | **Severino** | 0 | 1 | 0 |
| **Cavalante** | 0 | 1 | 0 | **Lucena** | 0 | 1 | 0 | **Silva** | 24 | 63 | 52 |
| **Cavalcante** | 0 | 1 | 1 | **Luna** | 0 | 1 | 0 | **Sobrinho** | 0 | 3 | 0 |
| **Conceicao** | 0 | 0 | 23 | **Maia** | 0 | 1 | 1 | **Socorro** | 0 | 0 | 1 |
| **Costa** | 1 | 9 | 4 | **Marcelino** | 0 | 1 | 0 | **Sousa** | 1 | 17 | 22 |
| **Dantas** | 1 | 3 | 3 | **Maria** | 0 | 1 | 0 | **Targino** | 0 | 1 | 0 |
| **Dias** | 0 | 0 | 1 | **Marines** | 0 | 0 | 1 | **Torquato** | 0 | 1 | 0 |
| **Diniz** | 2 | 8 | 6 | **Martins** | 0 | 1 | 0 | **Torres** | 0 | 2 | 2 |
| **Dores** | 0 | 0 | 1 | **Melo** | 0 | 6 | 3 | **Vespucio** | 0 | 1 | 0 |
| **Duarte** | 0 | 0 | 1 | **Mesquita** | 0 | 2 | 1 | **Vieira** | 0 | 1 | 3 |
|  |  |  |  | **Xavier** | 0 | 0 | 1 |  |  |  |  |

**FIGURE 1S -** Distribution of a = A/N (graph a) and d = D/N (graph b) according to human development index (HDI) of all countries and regions listed in Table I. Spearman's rank correlation coefficients (r) and their corresponding p-values are written above each graph.

**
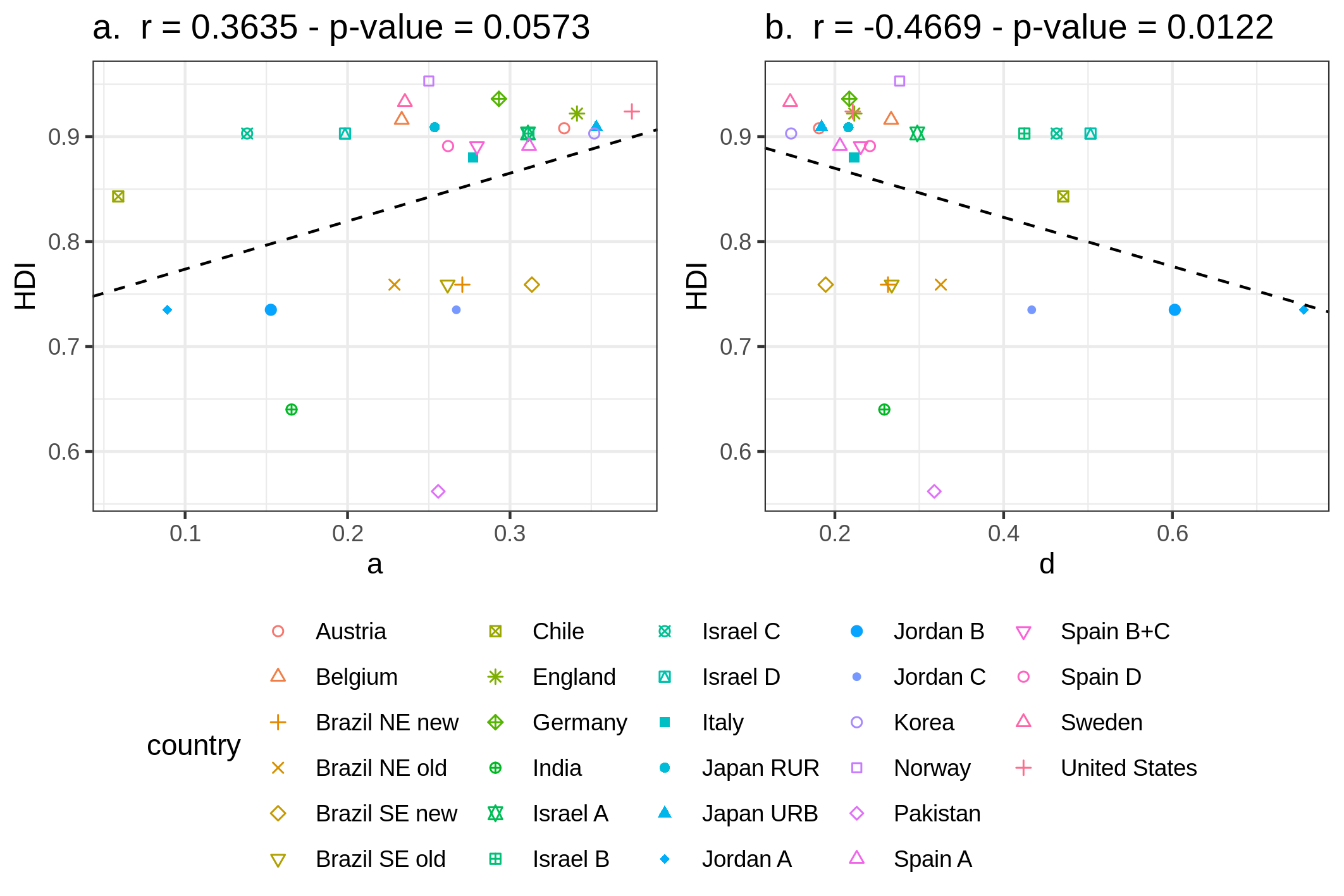
**
